# Multi-Objective Optimization of IME-Based Acoustic Tweezers for Mitigating Node Displacements

**DOI:** 10.3390/polym17152018

**Published:** 2025-07-24

**Authors:** Hanjui Chang, Yue Sun, Fei Long, Jiaquan Li

**Affiliations:** 1 Department of Mechanical Engineering, College of Engineering, Shantou University, Shantou 515063, China; 22ysun@stu.edu.cn (Y.S.); 23flong@stu.edu.cn (F.L.); 22jqli2@stu.edu.cn (J.L.); 2 Intelligent Manufacturing Key Laboratory of Ministry of Education, Shantou University, Shantou 515063, China

**Keywords:** acoustic tweezers, IME technology, multi-objective optimization, NSGA-II-MOPSO, micro/nano manufacturing, thermal–mechanical coupling, wave transmission efficiency

## Abstract

**Highlights:**

**What are the main findings?**

- Developed a novel IME-based microfabrication process for PMUTs with sub-micron alignment accuracy via thermal compression bonding.
- Proposed a hybrid NSGA-II-MOPSO optimization algorithm that simultaneously minimized acoustic node displacement (≤5.2 μm), volumetric shrinkage (≤0.8%), and residual stress (≤12 MPa).
- Established a multidisciplinary evaluation framework integrating FEM and in situ process monitoring for comprehensive PMUT performance assessment.
- Achieved a 27.3% reduction in node displacement amplitude and a 19.6% improvement in ultrasonic transmission uniformity compared to conventional fabrication methods.
- Demonstrated feasible integration of PMUT arrays with polymeric substrates using optimized injection molding parameters (100 MPa packing pressure, 250 °C melt temperature, 20 s packing time).

**What is the implication of the main finding?**

- The IME-based microfabrication process with sub-micron alignment advances precision in PMUT manufacturing, enabling more reliable miniaturized ultrasonic transducers for high-performance applications.
- The hybrid optimization algorithm addresses critical performance trade-offs, enhancing PMUT reliability and consistency by controlling key metrics (displacement, shrinkage, stress) within strict limits.
- The multidisciplinary evaluation framework improves design validation and manufacturing quality control for microdevices.
- Performance gains over conventional methods (reduced node displacement, better transmission uniformity) enhance PMUT efficiency for applications like medical imaging and sensing.
- Feasible integration with polymeric substrates expands PMUT applicability to flexible, low-cost platforms, enabling new use cases in wearable or disposable devices.

**Abstract:**

Acoustic tweezers, as advanced micro/nano manipulation tools, play a pivotal role in biomedical engineering, microfluidics, and precision manufacturing. However, piezoelectric-based acoustic tweezers face performance limitations due to multi-physical coupling effects during microfabrication. This study proposes a novel approach using injection molding with embedded electronics (IMEs) technology to fabricate piezoelectric micro-ultrasonic transducers with micron-scale precision, addressing the critical issue of acoustic node displacement caused by thermal–mechanical coupling in injection molding—a problem that impairs wave transmission efficiency and operational stability. To optimize the IME process parameters, a hybrid multi-objective optimization framework integrating NSGA-II and MOPSO is developed, aiming to simultaneously minimize acoustic node displacement, volumetric shrinkage, and residual stress distribution. Key process variables—packing pressure (80–120 MPa), melt temperature (230–280 °C), and packing time (15–30 s)—are analyzed via finite element modeling (FEM) and validated through in situ tie bar elongation measurements. The results show a 27.3% reduction in node displacement amplitude and a 19.6% improvement in wave transmission uniformity compared to conventional methods. This methodology enhances acoustic tweezers’ operational stability and provides a generalizable framework for multi-physics optimization in MEMS manufacturing, laying a foundation for next-generation applications in single-cell manipulation, lab-on-a-chip systems, and nanomaterial assembly.

## 1. Introduction

Early advancements in cell visualization techniques laid the foundation for the development of cell theory, while recent breakthroughs in single-cell and biomolecule manipulation have propelled progress in microbiology, molecular biology, biophysics, and bioanalytical chemistry [1]. Untethered robots at the millimeter and sub-millimeter scales have garnered increasing attention for their potential to revolutionize healthcare and bioengineering. As robot size shrinks to the single-cell order, previously inaccessible bodily sites become available for high-resolution in situ and in vivo manipulations [2]. Thus, the contactless capture and transport of microparticles, cells, bacteria, nanoparticles, and exosomes are essential for exploring the microscopic world [3]. Mechanical forces play pivotal roles in regulating biological processes at molecular and cellular levels—such as gene expression, adhesion, migration, and cell fate—critical for maintaining tissue homeostasis. Microengineering platforms that mimic in vivo micromechanical environments are vital for studying cellular processes in normal and pathophysiological contexts [4]. Acoustic waves, as mechanical waves carrying momentum and energy, induce an acoustic radiation force (ARF) on objects through absorption, scattering, and reflection [5]. Inspired by optical tweezers, acoustic tweezers leverage ARF in strong gradient acoustic fields to manipulate particles effectively. Acoustic tweezers represent a cutting-edge technology that uses ARF to capture and control tiny particles. Though acoustic waves have low energy, their radiation force per unit input can reach 100,000 times that of lasers. Acoustic waves—elastic waves—propagate through media like air [6], water [7], and biological tissues [8] without being affected by media transparency or electromagnetic properties. Their energy and frequency match medical ultrasound imaging systems, enabling the safe manipulation of single cells or nanoparticles. When a particle or cell enters the acoustic field, ARF “pushes” it to wave antinodes or nodes, where it is “locked.” Adjusting the acoustic source changes wave distributions to move the particle, while modifying field characteristics—based on particle-source correlations—enables sorting and classification [9]. The current research focuses on acoustic field design and manipulation mechanisms, such as standing-wave potential wells or single-beam focusing for particle capture. Recent progress has enabled 1D [10], 2D [11], and 3D [12] acoustic particle capture. Combining advanced acoustic techniques with precise controls allows multi-dimensional manipulation, opening new frontiers for micro/nanoscale particle handling. 3D acoustic tweezers hold particular promise in biomedicine for precise cellular localization and drug delivery, enhancing therapeutic efficacy. Hence, this study designs and optimizes 3D acoustic tweezers. In 2019, Omid Y Oussefi et al. [13] integrated magnetic and acoustic micromanipulation to achieve 3D, contactless, semi-autonomous micromanipulation with full automation potential for microassembly. They processed solid/liquid materials (300 μm to 3 mm) via acoustic levitation in a 30 mm × 4 mm cylindrical workspace, using external magnetic fields to control magnetically active components. In 2024, Teng Li et al. [14] developed chirality-tunable acoustic vortex tweezers, enabling chirality adjustment, biological barrier penetration, capture of micro-to-millimeter objects, and rotational control—with programmable robots enabling contactless high-resolution object translation. Piezoelectric micromechanical ultrasonic transducers (PMUTs) have emerged as superior alternatives to conventional piezoelectric composite transducers due to the advantages of microelectromechanical systems (MEMS), addressing assembly complexity, size, and weight issues [15]. PMUTs allow high-fill-factor piezoelectric transducer array fabrication from cavity SOI wafers via a simple triple-mask process, yielding smaller diameters (25 μm) and finer pitches than traditional through-wafer etching [16]. Piezoelectric micromechanical ultrasonic transducers (PMUTs), a type of diaphragm-like thin-film bending transducer typically formed on silicon substrates, are a potential solution for integrated transducer arrays [17]. Unlike bulk piezoelectric ceramic-based transducers, PMUTs feature flexible membrane structures for low acoustic impedance and superior acoustic coupling to air/liquids [18]. Therefore, in this study, we fabricated acoustic tweezers via injection molded in-mold electronics (IME) technology [19] to use PMUT arrays as circuit films for in-mold electronics decoration. With IME technology, we were able to embed the PMUT arrays precisely inside the mold to form a micrometer-sized decorative film. This technology not only provides acoustic tweezers with excellent maneuverability but also provides them with good durability and stability. The acoustic tweezers developed at our institute have the advantages of high manipulation precision, easy operation, and low cost and are suitable for a variety of micro- and nanoscale application scenarios. This study fabricates acoustic tweezers using injection-molded in-mold electronics (IMEs) technology, embedding PMUT arrays as circuit films for decorative IME layers. This approach endows tweezers with excellent maneuverability, durability, and stability, offering high precision, ease of operation, and low cost for diverse micro/nanoscale applications. Nodal displacement—a key performance metric in acoustic tweezer fabrication—directly impacts acoustic wave transmission and manipulation efficiency. Caused by improper injection molding parameters, the nodal displacement alters sound wave propagation paths and efficiency [20]. Optimizing molding parameters (e.g., holding pressure, melt temperature, holding time) to control nodal displacement is critical for enhancing tweezer performance [21]. Balancing multiple process parameters is essential for overall optimization. Traditional algorithms (e.g., single-objective optimization, genetic algorithms, particle swarm optimization) have limitations in multi-objective scenarios, necessitating a comprehensive literature review to select optimal methods. Section 2 summarizes the prior studies and compares optimization approaches. Section 3 details material selection, properties, methodologies, and formulas. Section 4 describes the experimental preparation—including acoustic tweezer model design, simulation steps, and molding parameter settings. Section 5 presents and analyzes results (nodal displacement, volumetric shrinkage, residual stress data/graphs). Finally, Section 6 concludes this study, discusses its significance, and outlines future directions.

## 2. Literature Review

In 2015, Sina Akhbari et al. [22] proposed a method to fabricate self-bending diaphragms on thin films via the engineering of residual stress for the construction of high-response piezoelectric micromechanical ultrasonic transducers. A 0.65 μm thick low-stress silicon nitride (SiN) film and a low-temperature oxidation (LTO) film were placed on a 4 μm thick silicon film to produce an ideal self-bending diaphragm.

In 2015, Yipeng Lu et al. [16] prepared and characterized two devices with different piezoelectric layers, lead zirconium titanate (PZT) and aluminum nitride (AlN). Compared with the PZT PMUTs with a diameter of 50 μm, the PZT PMUTs exhibit a dynamic displacement sensitivity of 316 nm/V in air at 11 MHz, which is approximately 20 times greater than that of the AlN PMUTs.

In 2021, Tony Jun Huang et al. [23] demonstrated acoustoelectronic nanotweezers (AENTs), which combine the advantages of electricity and acoustics to enable the massively parallel dynamic manipulation of particles less than 100 nanometers in size.

In 2023, Xuemei Ren et al. [24] proposed constructing a multifunctional acoustic tweezer that is capable of multiple particle manipulation functions in response to the inability to manipulate particles with a single holographic lens. The ability to realize the fixed-angle rotation of rectangular particles, multimodal particle alignment, selectable left-right movement of particles on a line, and the ability to complete two different trajectories with a single particle was demonstrated.

In 2023, Zeping Gao et al. [25] proposed an ultrasound phased array-based online structure assembly platform that integrates the precise selection, movement, rotation, and precise combination of organoids under different conditions for the structural construction of cisplatin-induced localized renal injuries and the cultivation of heterogeneous assemblies.

In 2017, Kenjiro Fukuda et al. [26] reviewed recent developments in printed thin-film transistors (TFTs) and integrated circuits in terms of materials, printing technologies, and applications.

However, resin injection is performed such that the polymer film tears in localized areas near the edges and corners, which has a significant effect on the properties and deformation of the polymer film [27].

In 2023, Xiaodong Chen et al. [28] analyzed the challenges of flexible sensing performance in real-world applications, summarized the issues of compatible sensor–biology interfaces, and briefly discussed the powering and connectivity of sensor networks.

To achieve stable acoustic wave transmission, the surface quality and performance of the printed circuit film are improved by optimizing the injection molding parameters when performing injection molding with IME technology. Thus, when the injection molding parameters are optimized, multiple optimization objectives are often faced, which makes traditional single-objective optimization algorithms no longer applicable. Therefore, we usually turn to several multi-objective optimization algorithms, such as nondominated sorting genetic algorithm II (NSGA II) [29] and multi-objective particle swarm optimization (MOPSO) [30]. Although the NSGA-II algorithm can effectively maintain the diversity and uniformity of the set of nondominated solutions, it has a slow convergence speed when dealing with high-dimensional problems and is prone to falling into local optimal solutions [31]. The MOPSO algorithm [32], on the other hand, has a better global search ability, but it easily falls into local optimal solutions during the solution space search, which leads to a slower convergence speed of the algorithm and makes it difficult to maintain the diversity of the population effectively.

To overcome the shortcomings of these algorithms, we propose a hybrid multi-objective optimization algorithm, NSGA-II-MOPSO, which combines the advantages of NSGA-II and MOPSO, and improves the efficiency and stability of the optimization process through the fast convergence of the NSGA-II algorithm and the global searching ability of the MOPSO algorithm. In the NSGA-II-MOPSO algorithm, we use the NSGA-II algorithm to maintain the diversity of the population and utilize the MOPSO algorithm to increase the global search ability to explore the solution space better and find a better set of Pareto-optimal solutions.

In 2014, Hanieh Borhanazad et al. [30] used a multi-objective particle swarm optimization (MOPSO) approach to find the optimal configuration of the system and size of the components.

In 2017, Narges Ghorbani et al. [32] applied the hybrid genetic algorithm with particle swarm algorithm (GA-PSO) to the optimal size problem.

In 2021, Hongwei Li et al. [31] proposed a fast system optimization method that combines ANOVA, agent modeling, and a nondominated sequential genetic algorithm (NSGA-II).

In 2020, Arunachalam Sundaram et al. [29] proposed a synergistic hybrid metaheuristic algorithm that combines a nondominated sorting genetic algorithm and a multi-objective particle swarm optimization algorithm. Compared with several existing algorithms, the hybrid algorithm can obtain better scaling and diversified Pareto-optimal solutions and converge to the actual Pareto-optimal frontier faster.

In 2023, Zhe Xu et al. [33] used a hybrid method of NSGA-II and MOPSO, i.e., NSGA-II-MOPSO, to solve multi-objective optimization problems. This hybrid method has the advantages of strong exploration to obtain better convergence and strong exploitation to eliminate local optima.

Since the injection molding process involves multiple variables and complex physical phenomena, the RPE method can provide robust performance evaluation results through statistical analysis and data processing to reduce the influence of experimental errors and improve the accuracy of the optimization results. Therefore, in the course of this paper, we adopt the recognizable performance evaluation (RPE) method to evaluate the performance of injection-molded acoustic tweezers. The RPE method is a widely used evaluation technique in multi-objective optimization problems, which measures the performance of the system through the signal/noise ratio (S/N ratio) performance of the system, thus providing explicit guidance for the optimization process.

In this study, we evaluate the nodal displacement, volumetric shrinkage, and residual stress of acoustic tweezers via the RPE method by optimizing key parameters such as the holding pressure, melt temperature, and holding time. Through the signal-to-noise ratio analysis, we identified the optimal combination of injection molding parameters and achieved a significant improvement in the performance of the acoustic tweezers.

In 2013, Pei Chee Yong et al. [34] proposed an improved a priori SNR estimator to overcome the delay. The evaluation is carried out by using objective evaluation metrics to measure the trade-off between noise reduction, speech distortion, and music noise in the enhanced signal.

In 2020, Chunyan Huang et al. [35] used classical Lena images, flower images, and Cameraman images for simulation experiments and evaluated the experimental results in terms of the signal-to-noise ratio (SNR) and peak signal-to-noise ratio (PSNR).

In 2020, Guoliang Shao et al. [36] verified the relationship between the measurement of signal-to-noise ratio and the distance estimation accuracy through simulation. In addition, based on the results of the signal-to-noise ratio evaluation, the selection strategy of the target magnet, the effective signal-to-noise ratio threshold of the localization algorithm, and the optimal distribution of the sensors are proposed.

In 2021, Han-Jui Chang et al. [37] proposed a screw life prediction method based on a hybrid composite screw process parameter method via a dynamic iterative approach. Additionally, a combined application of an automatic virtual metering system and a recognizable performance evaluation (RPE) program was proposed.

In 2022, Hanjui Chang et al. [38] investigated the relationship between kf and Kt from the perspective of virtual measurement (VM) and recognizable performance evaluation (RPM). These relationships were compared with the filling data to establish a crack initiation control model based on the anomalous filling pressure.

Therefore, to validate the optimization effect, we utilize an identifiable performance assessment method to comprehensively evaluate the optimization objectives. By evaluating key performance metrics such as nodal displacement, volumetric shrinkage, and residual stress, we can ensure that the optimized acoustic tweezers have excellent performance in particle capture and control.

In conclusion, the existing studies have explored acoustic manipulation, PMUT device design, injection molding process optimization, and multi-objective optimization methods from different perspectives, providing a theoretical basis and methodological support for this research. However, these efforts often focus on theoretical modeling, algorithm construction or device performance evaluation, lacking systematic research that combines injection molding with embedded acoustic components. The research in this paper is precisely based on this, focusing on the manufacturing accuracy control problem of PMUT-embedded acoustic tweezers in the injection molding process. It proposes to regulate the key process parameters through a multi-objective hybrid optimization algorithm and combine the identifiable performance evaluation method to achieve a comprehensive improvement in the performance of acoustic tweezers. This method provides a new technical path in terms of structural stability, consistency of sound wave propagation, and process controllability, laying the foundation for the practical application of acoustic control devices.

Based on the comprehensive analysis of the literature review mentioned above, the aim of this study is to develop an acoustic tweezer that utilizes the acoustic radiation force generated by acoustic waves in thin films to achieve the capture and control of particles. To construct a high-performance acoustic tweezer, a PMUT is used as the thin film, which is fabricated into a thin film form via in-mode electronics (IME). The construction of the acoustic tweezers will be realized by thermally compressing the PMUT film on the model. During the optimization of the acoustic tweezers, we use the NSGA-II-MOPSO multi-objective hybrid optimization algorithm to optimize the injection molding parameters to achieve the best performance of the tweezers in different environments and application scenarios. Ultimately, we will conduct a comprehensive evaluation of the optimization objectives via a recognizable performance assessment method to ensure that the acoustic tweezers have excellent performance in terms of particle capture and control.

**Figure 1:**
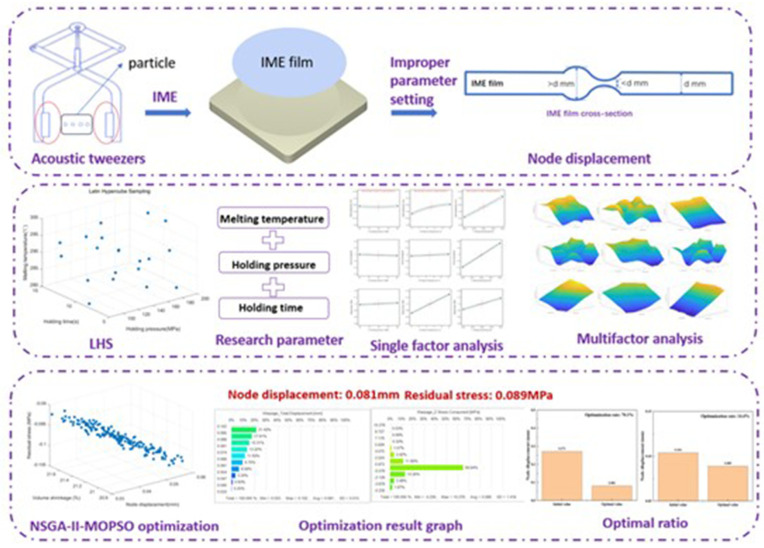
Acoustic tweezers optimization summary diagram.

As shown in Figure 1, this study aims to ensure the nodal displacement of IME films for acoustic tweezers by optimizing the injection molding parameters. By systematically adjusting and optimizing the injection molding parameters, we can effectively control the nodal displacement of IME films. We focus on adjusting the injection molding parameters, such as temperature, pressure, and time, to optimize the nodal displacement of the IME film maximally and ensure the stability and reliability of the acoustic tweezers.

Through experimental validation and numerical simulation analysis, we thoroughly explore the effects of different injection molding parameters on the nodal displacement of IME films and determine the optimal combination of injection molding parameters; this will provide an important reference for the design and fabrication of acoustic wave emitters and lay a solid foundation for their best performance in practical applications.

## 3. Materials and Methods

### 3.1. Materials

As a material for a PMUT, first, the material should have good piezoelectric properties, i.e., it should be able to produce a significant piezoelectric effect under the action of an applied electric field to realize the conversion of electrical energy to mechanical energy. Second, the material needs to have good flexibility and elasticity to adapt to the different morphologies and surface characteristics of the PMUT device and be able to produce reversible deformation under force. Finally, the preparation cost should be as low as possible, and the preparation process should be simple and controllable to improve the device preparation efficiency and reduce the production cost. The preparation of in-mold flexible circuits requires the selection of suitable polymer materials to meet their needs in different applications.

Polyether ester (PEEK) [39] is a polymer material that is highly considered in the selection process for in-mold flexible circuits. PEEK has excellent mechanical properties, high-temperature stability, and chemical stability, which makes it highly desirable in the field of in-mold flexible circuits. In contrast, other materials may fall short in certain areas.

Compared with polyimide (PI) [40], PEEK has greater strength and stiffness, thus providing better structural support and tensile strength in flexible circuits. In addition, PEEK has better heat resistance than does PI and is able to maintain stable performance at higher temperatures, making it suitable for applications in high-temperature environments. Compared with polyethylene terephthalate (PET), PEEK has better chemical and abrasion resistance and is able to maintain stable performance in more complex operating environments. In addition, PEEK has better electrical conductivity and is better able to meet the electrical conductivity requirements of flexible circuits. Compared with polyimide (PAI) [41], PEEK is more processable and cost-effective, can be more easily prepared into various shapes of flexible circuits, and has better electrical conductivity and chemical stability, which can be useful in a wider range of applications.

**Figure 2:**
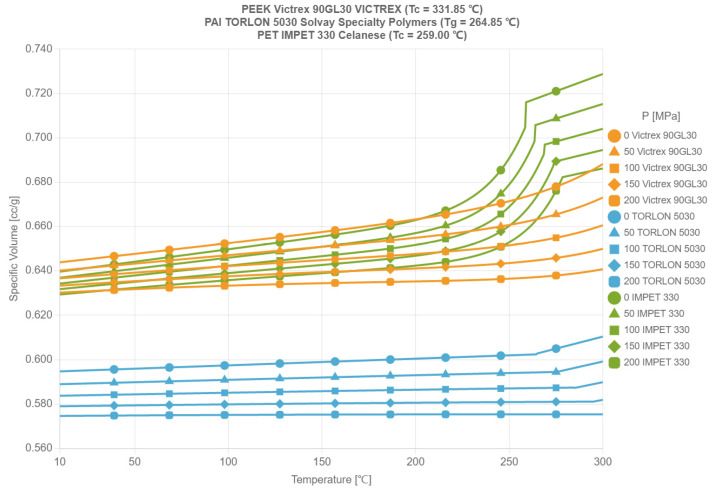
PVT comparison diagram of circuit film materials.

In summary, as shown in Figure 2, the PVT curves of the three materials are compared. PEEK has clear advantages as a material for in-mold flexible circuits, and its excellent mechanical properties, high-temperature stability, and chemical stability make it a highly favorable option.

### 3.2. Methods

#### 3.2.1. Latin Hypercube Sampling (LHS)

Latin hypercube sampling (LHS) is a statistical method used to generate representative sample points in a multidimensional parameter space. It ensures the efficient and uniform coverage of the parameter space by dividing each variable’s range into equal probability intervals and randomly selecting one value from each interval without replacement [42]. These sampled values are then combined randomly across variables to create unique sample points, ensuring balanced representation of the entire parameter space while maintaining independence between variables.

Compared to traditional sampling methods, Latin hypercube sampling (LHS) achieves better parameter space coverage with fewer samples, reducing computational costs. It is effective in high-dimensional spaces, supports various probability distributions, and is well-suited for sensitivity analysis and optimization, even when prior distribution knowledge is limited [43].

#### 3.2.2. NSGA II-MOPSO Optimization Method

In this study, we combine two stochastic multi-objective optimization algorithms, nondominated sorting genetic algorithm II (NSGA-II) and multi-objective particle swarm optimization (MOPSO), to optimize the injection molding parameters. To optimize the injection parameters, NSGA-II uses the principles of elitism, sorting, and congestion distance computation to increase the distributivity of the solutions while maintaining the diversity of Pareto optimal (PO) solutions. However, the crowded comparison of the NSGA-II algorithm may limit its convergence. In contrast, MOPSO’s particles do not use genetic operators, and its information sharing mechanism is different from that of NSGA-II. In MOPSO, nondominated solutions called leaders are utilized to guide other particles. These particles search the space by updating their velocity and inertia weights. For complex problems, MOPSO tends to fall into local optima, but this can be avoided by continuously updating the parameters of MOPSO.

**Figure 3:**
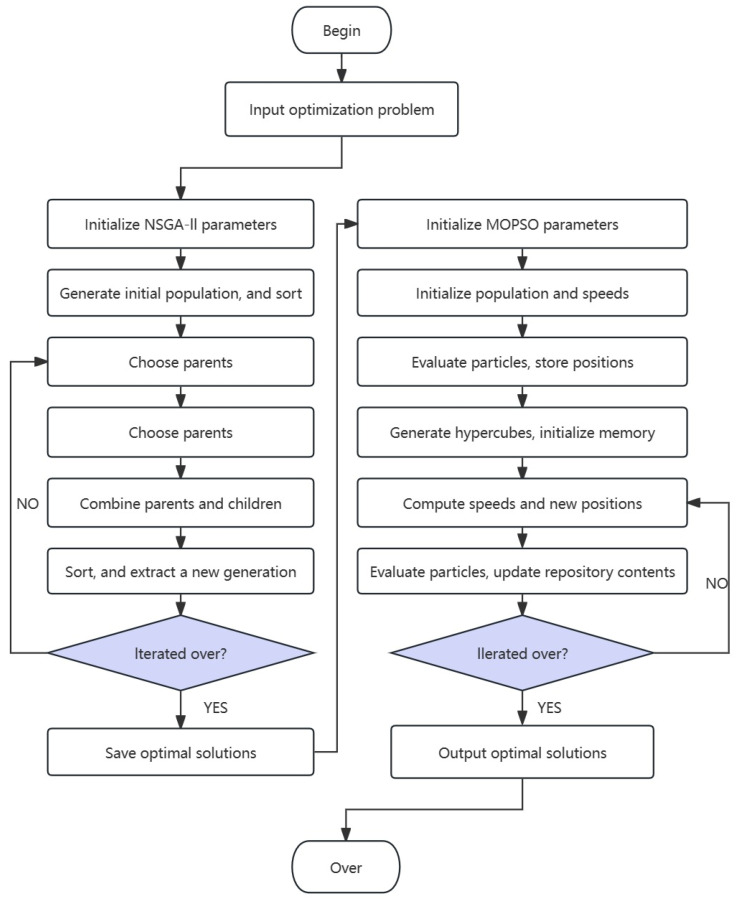
Flowchart of NSGA-II-MOPSO.

Combining the advantages of the NSGA-II and MOPSO algorithms, we achieve the following breakthroughs in this study. First, through the elitism and diversity maintenance of NSGA-II, we ensure a wide distribution of understanding. Second, through the leader mechanism and speed update of MOPSO, the depth search of the solution space is realized. In addition, combining the two algorithms enables us to overcome the limitations of the respective algorithms; NSGA-II tends to fall into local optimality, whereas MOPSO converges slowly, as shown in Figure 3. Combining the two can ensure the efficiency and convergence of the global search while maintaining diversity.

Based on the 20 sets of data obtained from the Latin hypercube sampling, we designed the optimization flow of NSGA-II-MOPSO for the three injection parameters of melt temperature, holding pressure, and holding time, as follows.

1. Initialization population: A set of initial solutions was randomly generated as the starting population of the NSGA-II-MOPSO algorithm. Each solution included a combination of three injection parameters: melt temperature, holding pressure, and holding time.

2. Adaptation evaluation: For each solution in the population, the data obtained from Latin hypercube sampling were used to simulate the injection molding, and the corresponding nodal displacement and volume shrinkage were calculated as the adaptation values of the two optimization objectives.

3. Combination of the NSGA-II and MOPSO algorithms: Two algorithms, NSGA-II and MOPSO, were combined to form a hybrid optimization algorithm. In NSGA-II, nondominated sorting and congestion distance computation were utilized to maintain solution diversity; in MOPSO, a deep search of the solution space was achieved through a leader mechanism and particle updating.

4. Nondominated ordering and Pareto frontiers: Nondominated ordering of solutions in the population and delineation of different Pareto fronts via the NSGA-II algorithm.

5. Multi-objective Particle Swarm Optimization: In the MOPSO algorithm, multiple leader particles are used to guide other particles to search the solution space. The two objectives of node displacement and volume shrinkage are continuously optimized by updating the velocity and position of the particles.

6. Update Population: Based on the results of NSGA-II and MOPSO, the solutions in the population were updated. The diversity and convergence of the population were maintained through genetic manipulation and particle updating.

7. Termination condition detection: Detect whether the node displacement and volume shrinkage satisfy the termination condition.

8. Output optimization results: Finally, a set of optimal solutions on the Pareto front was obtained, where each solution represented an optimal combination of injection parameters for node displacement and volume shrinkage.

#### 3.2.3. Multiple Recognizable Performance Evaluation

Multiple recognizable performance evaluation methods aim to combine multiple performance metrics to comprehensively evaluate the performance of a system. The principle is to determine the performance of the system in different aspects by combining multiple performance evaluation metrics, such as accuracy, speed, and robustness. The application of this method to evaluate the injection molding optimization of acoustic tweezers and it can provide multiple perspectives on system performance and provide a more comprehensive basis for optimization.

In this method, the experimental results are transformed via the signal-to-noise ratio rather than the mean value to evaluate the quality of the eigenvalues. The signal-to-noise ratio (SNR) represents the ratio between the signal and the background noise, and a higher SNR means that the signal is more pronounced relative to the noise; therefore, the eigenvalues are more reliable. In the assessment of the injection optimization of acoustic tweezers, the SNR of each eigenvalue can be calculated by using the acoustic signal as the signal and the background noise in the experiment as the noise.

Based on this multiple recognizable performance evaluation method, the SNRs of different eigenvalues can be combined to determine which eigenvalues are more representative and reliable. By comparing the SNRs of different eigenvalues, the eigenvalues that have the greatest impact on system performance can be found and provide an important basis for system optimization.

First, several evaluation indices, nodal displacement and volume shrinkage, are set for the key performance parameters of the acoustic tweezers, namely, the melting temperature, holding pressure, and holding time. Then, the performance data under different parameter combinations are obtained through simulation.

Using multiple recognizable performance evaluation methods, we can analyze these data in a comprehensive way. By weighing the various indicators, the degree of influence of different parameter combinations on the performance of the acoustic tweezers can be derived. For example, a certain set of parameters may be effective in improving accuracy but perform poorly in terms of speed or robustness, whereas another set of parameters may improve speed and robustness but slightly decrease accuracy.

Finally, combined with a comprehensive evaluation of the different parameter combinations, we can determine the optimal combination of injection parameters to maximize the performance of acoustic tweezers. In this way, multiple recognizable performance evaluation methods can help us understand the performance characteristics of acoustic tweezers more comprehensively and provide a reliable basis for their optimization.

In the optimal parameter analysis, the experimental results are transformed via the signal-to-noise ratio instead of the mean value to evaluate the eigenvalues. The S/N ratio is calculated as follows:

(1) $$\frac{S}{N}=-10log(MSD)$$

where *MSD* is the mean square deviation of the output characteristics.

In the analysis of engineering quality characteristics, the signal-to-noise ratio can be classified into three categories: “The larger, the better”, “the smaller, the better”, and “the target value is the best”. The optimization objective of this study was to minimize node displacement, warpage, and residual stress in order to obtain an acoustic tweezers structure with excellent performance. Therefore, the signal-to-noise ratio type of “the smaller, the better” was uniformly adopted for analysis and calculation. These three quality characteristics can be expressed in *MSD* as follows:

(2) $${MSD}_{SIB}=\frac{1}{n}\sum_{i=1}^{n}y_{i}^{2}$$

where $y_{i}$ is the measured value of the ith sample and *n* is the total number of samples. Since −log is a monotonically decreasing function, the *S*/*N* value should be maximized. Therefore, the *S*/*N* value is calculated via Equation (2).

## 4. Case Study

**Figure 4:**
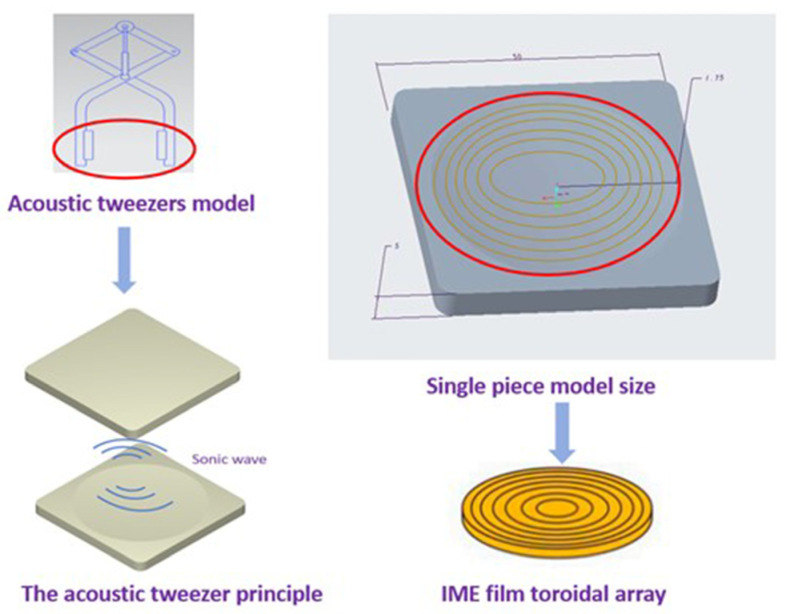
Acoustic tweezers 3D design.

In the experimental design, we first carried out the three-dimensional model design of the acoustic tweezers. As shown in Figure 4, to facilitate the use of tweezers to pick up particles, the model was designed to mimic the tweezers, and two parallel and concave faces were set in the clamping part, 50 mm × 50 mm × 5 mm. In the follow-up simulation experiment, we mainly studied this part and covered the two parts with a PMUT film as the sound field emission source.

We evaluated the structure and performance of acoustic tweezers by comparing different models, including one mold with one, two, and four cavities. The analysis results showed that the one-mold four-cavity design exhibited better uniformity during the plastic flow process, which was conducive to improving the molding quality and consistency of the product. Although it can be seen from Table 1 that the volume shrinkage rate of this structure is relatively large, due to the more significant impact of warpage on the shape accuracy and functional stability of the acoustic tweezers device, the advantage of the one-mode four-cavity in warpage control became a decisive factor. Therefore, after comprehensively considering various molding quality indicators, we chose the one-mold four-chamber structure as the final solution to achieve better structural stability and finished product performance.

Therefore, we next used the IME technique to cover the PMUT film inside the mold cavity for injection molding of the clamped portion. PMUT films are acoustic field-emitting materials that work based on the piezoelectric effect. When an electric field is applied, the piezoelectric material deforms and generates a mechanical vibration, which in turn generates sound waves in the surrounding medium. By adjusting the voltage applied to the PMUT, the frequency of vibration of the film can be controlled. This frequency adjustability allows the acoustic tweezers to adapt to sound field requirements at different frequencies, thus increasing the flexibility and versatility of their applications. In the structural design of PMUT films, the uniformity and propagation efficiency of the acoustic field need to be considered. Therefore, IME technology is used to cover the film on the plastic part, in which we need to optimize the parameters of the injection molding process to ensure the quality and functionality of the film.

The melt temperature is the temperature at which the plastic is heated to a molten state during the injection molding process. The proper melt temperature ensures that the plastic is completely melted and has good flow, which helps fill the mold cavity and reduces bubbles and defects.

The holding pressure is the pressure applied to the molten plastic while keeping the mold closed during the injection molding process. An appropriate holding pressure ensures that the plastic fully fills the mold cavity and reduces the generation of air bubbles and short stroke phenomena, thus improving the denseness and surface quality of the molded part.

The holding time is the length of time that the mold remains closed during the injection molding process. An appropriate holding time can ensure that the plastic in the mold cavity fully flows and fills, is conducive to the elimination of air bubbles, and improves the density of the molded part.

Next, we simulate the entire injection molding process in detail. Then, we simulate the entire injection molding process via Moldex3D software (R2024 version https://www.moldex3d.com, accessed on 4 November 2024). In addition, we also carry out parameter optimization iterations to obtain the optimal parameters via MATLAB 2022 software. By comparing the quality results obtained from the initial parameters and the optimized parameters, we summarize the analysis. In our research, we also focused on the application of tie bar elongation and pressure sensors during injection molding, which directly affect the control of pressure in the mold and the final quality of the injection parts.

## 5. Results and Discussion

To explore the optimization objectives, we considered various aspects of piezoelectric ultrasonic array films during the IME injection molding process. Among them, we paid special attention to the effects of nodal displacement and volume shrinkage on the signal emission performance.

First, nodal displacement directly affects the vibration of piezoelectric ultrasonic array films. During the IME injection molding process, the injection and filling of plastic materials affect the vibration characteristics of the film, which in turn affect the acoustic wave emission. Therefore, by analyzing the variation in nodal displacement, the vibration response of the film can be more accurately understood to optimize the injection parameters and improve the efficiency and stability of acoustic wave emission. Nodal displacement is also closely related to the structural stability and durability of the film. During the IME injection molding process, the flow and filling of the plastic generate certain stresses and deformations, which in turn lead to the deformation and displacement of the film. If the nodal displacement is too large or uneven, it may lead to instability of the film structure or even trigger film fracture or fatigue damage, thus affecting the performance and stability of the acoustic emission.

Second, volumetric shrinkage directly affects the dimensional and morphological changes of the film. During the IME injection molding process, the filling and cooling of the plastic material leads to changes in the volume of the film, thus affecting its structure and morphology. If the volumetric shrinkage is too large or uneven, uneven shape changes in the film may occur, which in turn affects the emission and propagation of sound waves. Volume shrinkage also affects the mechanical properties and stability of the film. Excessive volume shrinkage may lead to cracks or deformation on the surface of the film, which may affect its vibration characteristics and the stability of signal transmission.

Material properties and other relevant factors are taken into account when selecting the range of holding pressures, holding times, and melt temperatures. Melt temperatures in the range of 280 to 300 °C are chosen based on the melting point of the material used to ensure that the material maintains good flow and processability during the injection molding process. The holding pressure is set in the range of 80–200 MPa, mainly based on the flow and shrinkage characteristics of the material. Holding times between 5 and 15 s are chosen to consider the cooling rates and curing times of different materials, with shorter holding times for thinner molded parts and longer holding times for thicker parts to ensure full curing and strength [44].

**Figure 5:**
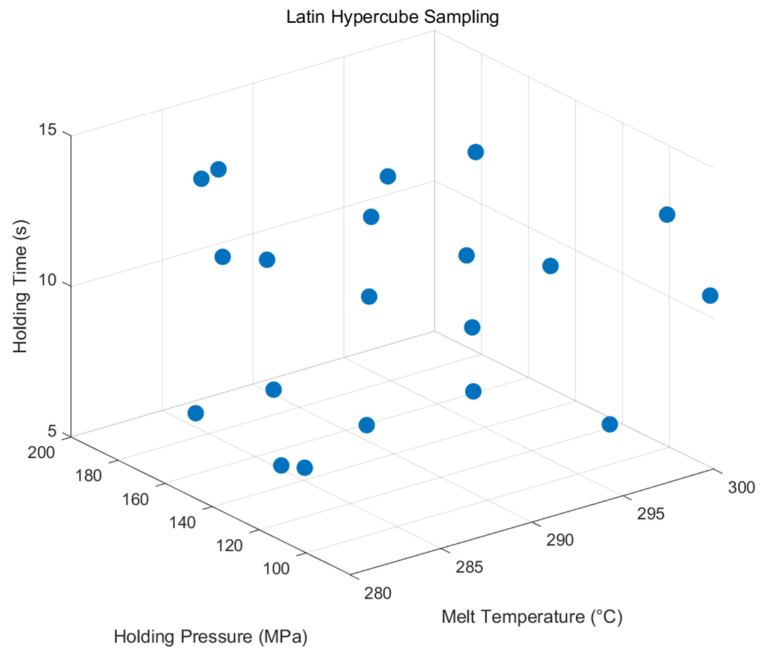
LHS sampling data.

Next, we perform Latin hypercube 3D sampling to ensure that the samples are uniformly selected across intervals of holding pressure, holding time, and melt temperature. Latin hypercube sampling divides each dimension into several uniform intervals to ensure that each interval has at least one sample point, thus effectively reducing the number of sample points while still ensuring the representativeness of the samples. We control for the following ranges of these three parameters: holding pressure in the interval (80, 200), holding time in the interval (5, 15), and melt temperature in the interval (280, 300). Within these ranges, we take 20 sets of data to obtain the sample distribution graph in Figure 5 to prepare for the next optimization experiments.

The 20 groups of data sampled from LHS were simulated, and Moldex3D (R2024 version https://www.moldex3d.com, accessed on 4 November 2024.) was adopted for simulation here. The results are shown in Table 2. The average joint displacement is 0.263 mm, the average volume shrinkage is 12.986%, and the average residual stress is 0.100 MPa.

### 5.1. Single Factor Influence Analysis

Based on 20 sets of data from LHS, three target values of node displacement, volume shrinkage, and residual stress are obtained. Next, we analyze and plot the data.

**Figure 6:**
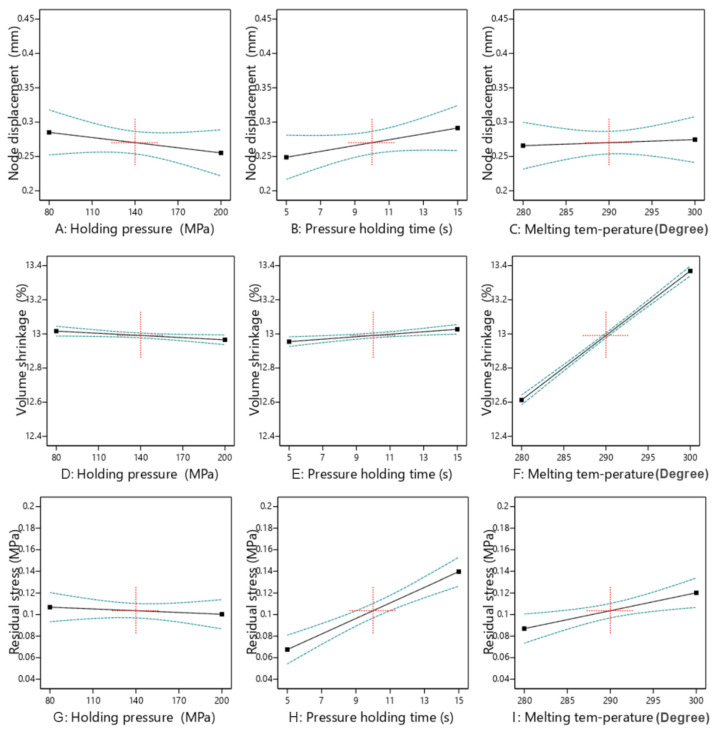
Influence of three factors on three goals.

By analyzing the single-factor effects of holding pressure, holding time, and melting temperature, Figure 6 is obtained, which shows the trends of how these factors act on nodal displacement, volumetric shrinkage, and residual stress.

Regarding nodal displacement, the holding pressure shows a trend of first decreasing and then increasing. An appropriate pressure is beneficial for reducing deformation, while an excessively high pressure will increase the material stress. The longer the holding time, the gradually increasing nodal displacement, and delayed pressure holding may lead to the accumulation of internal stress. The influence of the melting temperature is small, and the curve is generally gentle. Therefore, an appropriate holding pressure should be maintained, a relatively short holding time should be controlled, and an excessively high melting temperature should be avoided to reduce nodal deformation.

In terms of volumetric shrinkage, an increase in holding pressure slightly reduces the shrinkage rate, which is beneficial for mold filling and suppressing shrinkage. The extension of the holding time slightly increases the shrinkage rate, possibly due to the long cooling time of the material and insufficient stress release. The melting temperature has the most significant influence; an increase in temperature greatly increases the shrinkage rate because it enhances the fluidity of the material and causes greater volume changes. Therefore, a lower melting temperature, an appropriate holding pressure, and a short holding time should be selected to control shrinkage.

From the perspective of residual stress, an increase in holding pressure slightly reduces the stress, but the overall change is small, and the influence is relatively weak. The extension of the holding time increases the stress; the influence of the melting temperature is significant, and an increase in temperature greatly increases the stress.

In summary, the melting temperature has a significant influence on the three factors, and a high temperature increases the values of these three parameters. The holding pressure has a great influence on nodal displacement and residual stress but a small influence on volumetric shrinkage. When optimizing injection molding parameters, the selection of melting temperature and holding time should be focused on to achieve the goal of reducing nodal displacement, volumetric shrinkage, and residual stress.

### 5.2. Multifactor Influence Analysis

After completing the one-factor impact analysis, we further perform a two-factor impact analysis to more fully understand the effect of the interaction of each parameter on nodal displacement, volumetric shrinkage, and residual stress. Two-factor impact analysis helps reveal the combined effect on the performance metrics when both factors are varied simultaneously.

**Figure 7:**
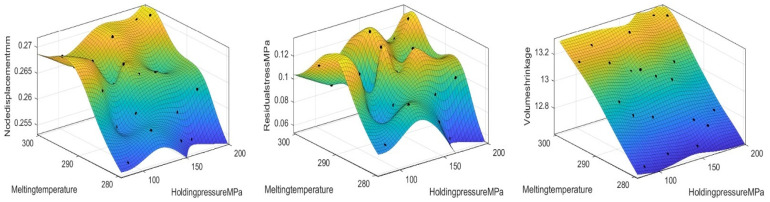
Response surface diagrams of the effects of the holding pressure and melting temperature on the node displacement, volume shrinkage rate, and residual stress.

As shown in Figure 7, the analysis reveals that the nodal displacement decreases with a decreasing holding pressure and melting temperature. The residual stress tends to decrease with a decreasing holding pressure and melting temperature. There is a significant reduction in volumetric shrinkage with a decreasing melt temperature. In addition, it is very little affected by the holding pressure. Therefore, to reduce the target value, we can reduce the holding pressure and melting temperature appropriately.

**Figure 8:**
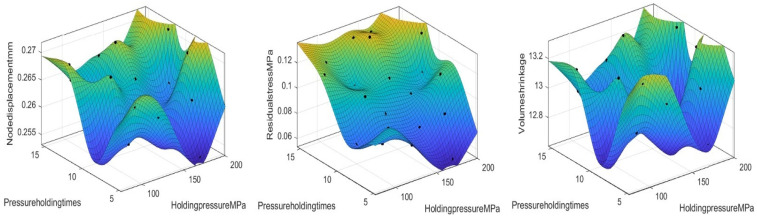
Response surface diagrams of the effects of the holding pressure and holding time on the node displacement, volume shrinkage rate, and residual stress.

Figure 8 shows the response surface plots of the effects of holding pressure and holding time on nodal displacement, residual stress, and volumetric shrinkage. The analysis revealed that volumetric shrinkage decreases with decreasing holding pressure and holding time. However, the nodal displacement as well as the volumetric shrinkage need suitable values, so the optimal values can be further searched in the following.

**Figure 9:**
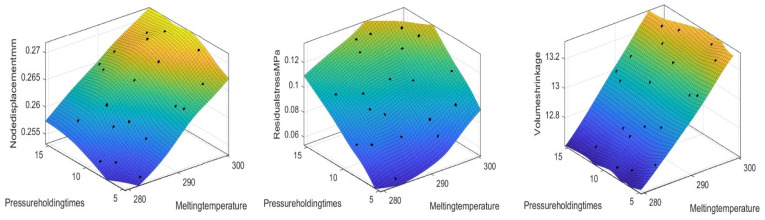
Response surface diagrams of the effects of the melting temperature and holding time on the node displacement, volume shrinkage rate, and residual stress.

Figure 9 presents response surface plots depicting how melt temperature and holding time affect nodal displacement, residual stress, and volumetric shrinkage. Analysis shows a lowering melt temperature and holding time reduces nodal displacement and residual stress. Volumetric shrinkage declines with decreasing melt temperature, while holding time has a weaker impact on it.

Through single-factor and two-factor analyses, we clarified the influences of holding pressure, melt temperature, and holding time on the three responses. Next, we will use the NSGA-II-MOPSO hybrid multi-objective optimization algorithm to further optimize injection molding parameters. This aims to balance nodal displacement, residual stress, and volumetric shrinkage optimally, enhancing the performance and stability of acoustic tweezers.

### 5.3. NSGA-II-MOPSO Multi-Objective Optimization

Based on these data, we conducted experiments and recorded the corresponding nodal displacements, volumetric shrinkage, and residual stresses. By performing multiple regression analysis on the collected data, we obtained the following fitting equations to describe the effects of each parameter on nodal displacement, volumetric shrinkage, and residual stress. The fitting equations for nodal displacement (*D*), volumetric shrinkage (*V*), and residual stress (*S*) are as follows:

$$D=78.2+0.03125x_{1}-0.5082x_{2}-0.8067x_{3}+6.64\times 10^{-7}x_{1}^{2}-2.724\times 10^{-3}x_{2}^{2}+2.776\times 10^{-3}x_{3}^{2}\;+7.474\times 10^{-5}{x_{1}x}_{2}-2.184\times 10^{-4}{x_{1}x}_{3}+3.707\times 10^{-3}{x_{2}x}_{3}\;+1.02\times 10^{-8}x_{1}^{3}-3.151\times 10^{-5}x_{2}^{3}-3.179\times 10^{-6}x_{3}^{3}\;+9.748\times 10^{-8}x_{1}^{2}x_{2}-1.993\times 10^{-8}x_{1}^{2}x_{3}+2.551\times 10^{-6}x_{2}^{2}x_{1}\;+1.119\times 10^{-5}x_{1}^{2}x_{2}+3.943\times 10^{-7}x_{3}^{2}x_{1}-6.71\times 10^{-6}x_{3}^{2}x_{2}\;-5.326\times 10^{-7}{x_{1}x}_{2}x_{3}$$

$$V=409.8-1.121x_{1}-5.682x_{2}-3.545x_{3}+5.225\times 10^{-4}x_{1}^{2}+0.1363x_{2}^{2}+0.01038x_{3}^{2}\;-3.926\times 10^{-3}{x_{1}x}_{2}+7.337\times 10^{-3}{x_{1}x}_{3}+0.03123{x_{2}x}_{3}\;-2.418\times 10^{-7}x_{1}^{3}-2.291\times 10^{-4}x_{2}^{3}-9.858\times 10^{-6}x_{3}^{3}\;-1.169\times 10^{-6}x_{1}^{2}x_{2}-1.416\times 10^{-6}x_{1}^{2}x_{3}-3.499\times 10^{-5}x_{2}^{2}x_{1}\;-4.298\times 10^{-4}x_{1}^{2}x_{2}-1.221\times 10^{-5}x_{3}^{2}x_{1}-4.23\times 10^{-5}x_{3}^{2}x_{2}\;+1.724\times 10^{-5}{x_{1}x}_{2}x_{3}$$

$$S=-353-0.6128x_{1}-0.4348x_{2}+3.992x_{3}-9.284\times 10^{-5}x_{1}^{2}+0.02679x_{2}^{2}-0.01492x_{3}^{2}\;+6.35\times 10^{-4}{x_{1}x}_{2}+4.339\times 10^{-4}{x_{1}x}_{3}+7.018\times 10^{-4}{x_{2}x}_{3}\;+5.408\times 10^{-8}x_{1}^{3}+1.33\times 10^{-4}x_{2}^{3}+1.844\times 10^{-5}x_{3}^{3}\;+5.321\times 10^{-7}x_{1}^{2}x_{2}+2.158\times 10^{-7}x_{1}^{2}x_{3}-2.065\times 10^{-5}x_{2}^{2}x_{1}\;-9.636\times 10^{-5}x_{1}^{2}x_{2}-7.567\times 10^{-6}x_{3}^{2}x_{1}+2.746\times 10^{-6}x_{3}^{2}x_{2}\;-1.35\times 10^{-6}{x_{1}x}_{2}x_{3}$$

Among them, *x*_1_ represents the holding pressure, with the unit of MPa; *x*_2_ represents the holding pressure time, with the unit of s; and *x*_3_ represents the melting temperature, with the unit of °C.

After obtaining the fitted equations for nodal displacement, volumetric shrinkage, and residual stress, we used these equations to perform an iterative optimization of NSGA-II-MOPSO for multi-objective optimization under the influence of three factors: holding pressure, melting temperature, and holding time.

**Figure 10:**
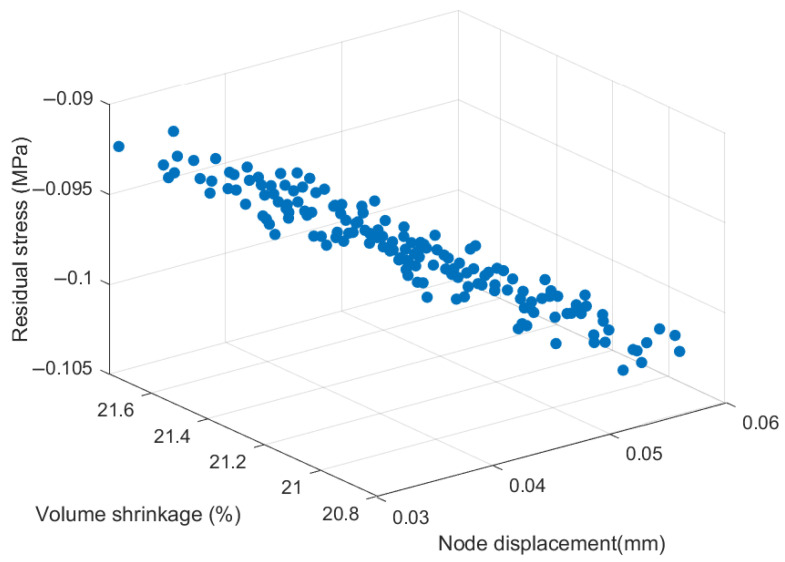
Pareto solution set for three object values under the NSGA-II-MOPSO method.

By iterating the multi-objective optimization through NSGA-II-MOPSO, we obtain the Pareto-optimal solution sets, as shown in Figure 10. These solution sets represent the trade-offs between nodal displacement, volumetric shrinkage, and residual stress for different combinations of parameters in the injection molding process. Each solution is not completely dominated by the other, resulting in a Pareto front that demonstrates multiple possible optimization schemes.

Within this set of Pareto-optimal solutions, a set of optimal parameter combinations was selected for further validation and analysis. Specifically, this set of optimal parameters includes a moderate holding pressure, melt temperature, and holding time to minimize volumetric shrinkage while maintaining low nodal displacement and residual stress. This set of parameters is chosen based on its balance and superiority in multi-objective optimization, which can achieve better coordination among the objective values.

Next, we validated and analyzed the selected optimal parameter combination in the Moldex3D (R2024 version) simulation software. Through this simulation, the effects of the optimized parameters on factors such as node displacement, residual stress, and volume shrinkage were thoroughly examined.

After the simulation verification of the optimal parameter combinations, we further evaluated the three objective values via the identifiable assessment method. As an innovative performance evaluation tool, the identifiable assessment method can provide more detailed analysis and insight into multi-objective optimization to ensure that the selected parameter combinations are efficient and reliable in practical applications.

**Figure 11:**
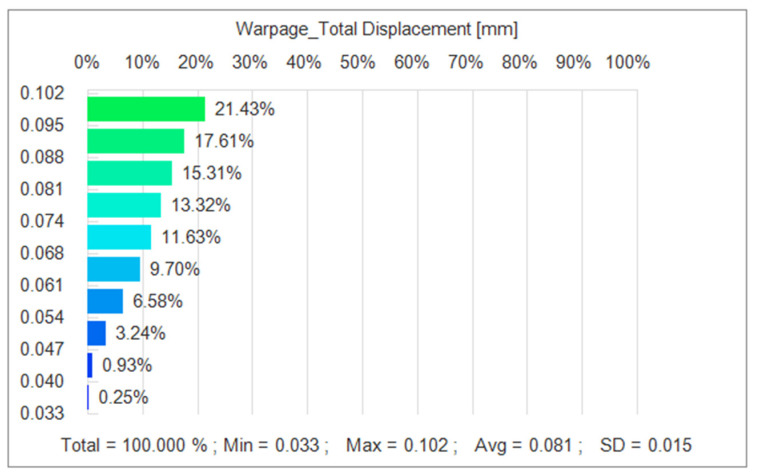
Node displacement after NSGA-II-MOPSO optimization.

In performing the iterative optimization of NSGA-II-MOPSO, we obtained the optimal combination of injection molding parameters. These parameters perform well in the simulation, and through further simulations, we analyzed the distribution of nodal displacements of the PMUT films. The simulation results are shown in Figure 11. Under the optimal parameter combination, the average node displacement is 0.081 mm, and this value is significantly lower than the node displacement under the initial design parameters, indicating the effectiveness of the optimization strategy. Further simulation reveals that the node displacement distribution is more uniform, indicating that the thermal and mechanical stresses are uniformly distributed during the injection molding process, which reduces the stress concentration and deformation inhomogeneity.

**Figure 12:**
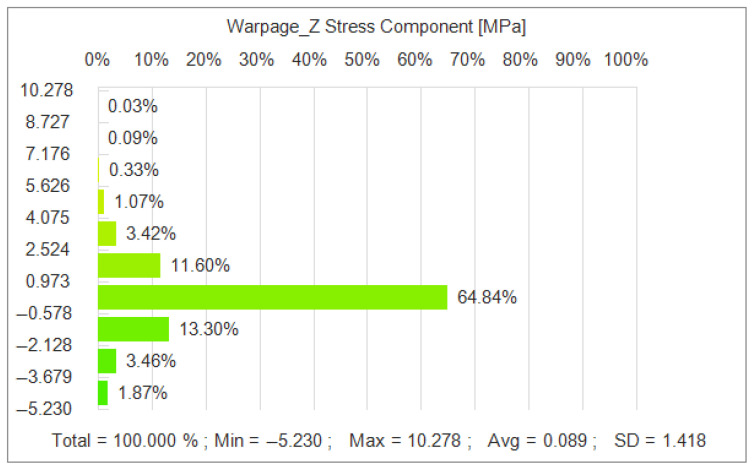
Residual stress distribution after NSGA-II-MOPSO optimization.

As shown in Figure 12, the optimized parameter combination also effectively reduces the residual stress generated during the injection molding process. The simulation results show that the residual stress is significantly reduced under the optimal parameters, and the overall average residual stress reaches 0.089 MPa. In comparison, under the initial parameter conditions, the residual stress is 0.100 MPa.

**Figure 13:**
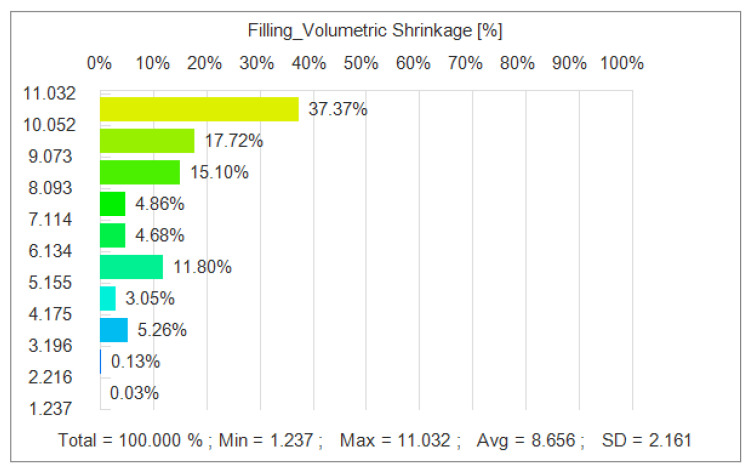
Volumetric shrinkage distribution after NSGA-II-MOPSO optimization.

In addition, as shown in Figure 13, the optimized parameter combination effectively reduces the volume shrinkage generated during the injection molding process. The simulation results show that the residual stresses are significantly reduced under the optimal parameters, and the overall average volumetric shrinkage reaches 8.656 MPa. Through simulation, we find that the optimized injection molding process significantly reduces the warpage and volumetric shrinkage phenomena and improves the surface quality of the film.

**Figure 14:**
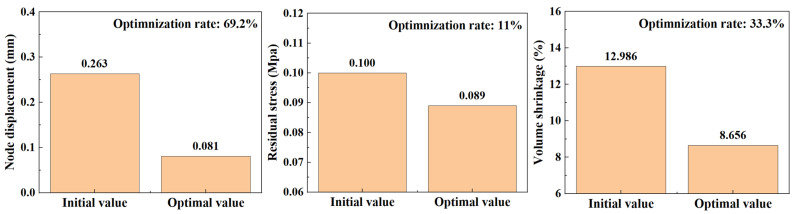
Optimization ratio chart.

In the iterative optimization of NSGA-II-MOPSO, we obtained the optimal parameter combinations and simulated the optimized nodal displacements via simulation software. After optimization, the average nodal displacement reaches 0.081 mm compared with 0.271 mm for the initial parameter conditions. This shows that through optimization, we reduce the nodal displacement by 0.182 mm, which is an optimization of approximately 69.2%. The average residual stress after optimization reached 0.089 MPa, which is an 11% reduction in residual stress compared with 0.100 MPa in the initial parametric condition. The optimized average volumetric shrinkage reached 8.656%, whereas it reached 12.986% in the initial parametric condition; the volumetric shrinkage was reduced by 33.3%. The optimized histograms of the average nodal displacement, residual stress, and volumetric shrinkage are shown in Figure 14.

These optimization results fully demonstrate the effectiveness of the NSGA-II-MOPSO multi-objective optimization algorithm in enhancing the performance of acoustic tweezers. The significant reduction in nodal displacement after optimization indicates that the acoustic tweezers are able to provide more accurate and stable acoustic wave transmission when capturing and manipulating particles. In addition, the reduction in residual stresses indicates a more uniform stress distribution during the injection molding process and improved structural integrity of the molded part. The significant reduction in volumetric shrinkage further indicates that the optimized combination of injection molding parameters can effectively control the deformation of the molded part, improving the dimensional accuracy and quality stability of the product.

### 5.4. Performance Evaluations

**Figure 15:**
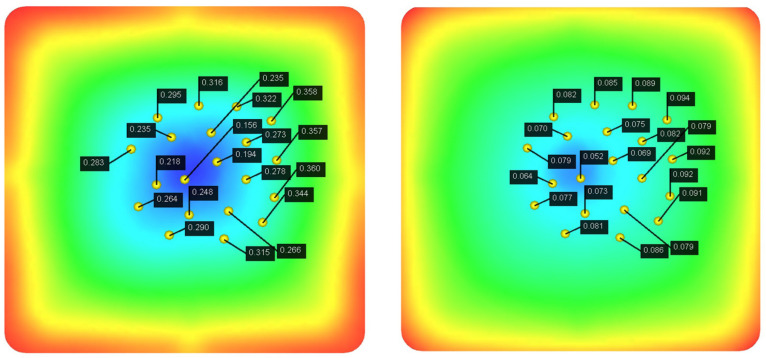
The 20 groups of node displacement before and after optimization.

Figure 15 shows a schematic diagram of the node displacement before and after optimization. To compare the node displacement before and after optimization, we used the identifiable performance evaluation method to analyze the node displacement.

Using the above 20 sets of node displacement values, we need to calculate the mean square error (*MSD*) of the output characteristics, which is expressed as follows:

$${MSD}_{SIB}=\frac{1}{n}\sum_{i=1}^{n}y_{i}^{2}$$

Plugging in the formula, we obtain *MSD*1 = 0.0816 and *MSD*2 = 0.006433

Next, according to the definition of SNR:

$$\frac{S}{N}=-10log(MSD)$$

For node displacement, the smaller the SNR is, the better. Therefore, the SNR of the optimized node displacement can be calculated in the following ways.

The obtained *MSD* is brought into the formula, and its corresponding signal-to-noise ratio is calculated according to the steps previously outlined.

$$\frac{S}{N}=-10\log\left(MSD1\right)=10.88$$

$$\frac{S}{N}=-10\log\left(MSD2\right)=21.92$$

The signal-to-noise ratio of the nodal displacement before optimization was 10.88 dB, which improved to 21.92 dB after optimization. This finding shows that the optimization measures significantly improved the signal-to-noise ratio of the nodal displacement data.

In the analysis of the experimental data, we first calculate the mean square error and signal-to-noise ratio before optimization. The low signal-to-noise ratio before optimization indicates that there is considerable noise interference in the data, resulting in instability and poor consistency of the output characteristics. We introduce a specific optimization method and adjust the design of the wire and the experimental conditions. Optimization methods include the selection of high-purity wire materials, precise control of the experimental ambient temperature and humidity, and the use of high-precision measurement equipment.

After optimization, we rerun the experiment and calculate the new mean square error and signal-to-noise ratio. After optimization, the mean square error is significantly reduced, resulting in a significant increase in the signal-to-noise ratio, which reaches 21.92 dB. A higher signal-to-noise ratio indicates that the optimized data quality has been significantly improved, the output characteristics are more stable, and the noise impact is significantly reduced.

By comparing the signal-to-noise ratios before and after optimization, we can clearly see the effectiveness and feasibility of the optimization method. After optimization, the signal-to-noise ratio is improved, which proves that the optimization method plays a significant role in improving the output characteristics of the wire. This result not only verifies the correctness of the theoretical analysis but also provides an important experimental basis and practical guidance for future circuit design and wire optimization.

**Figure 16:**
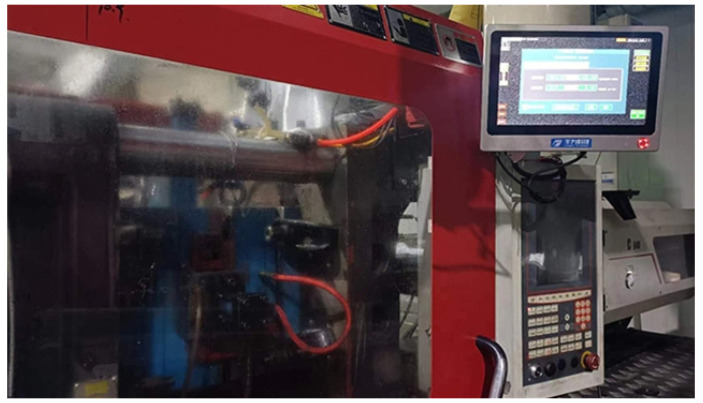
Schematic diagram of tie bar elongation during the clamping phase.

As shown in Figure 16, during the injection molding process, the force for clamping the mold is transmitted through the pull rod. The elongation of the pull rod reflects the clamping degree of the mold and the pressure inside the cavity. Its function is to connect the fixed part of the mold with the moving part and keep the mold closed to prevent plastic from overflowing and ensure uniform pressure distribution within the mold cavity.

In the injection molding machine operation, tie bars (tie-bars) are used to clamp the mold and ensure that the mold remains stable during the injection process. Strain sensors on the tie bar are used to measure the clamping force in real time. These sensors convert the pressure signal by detecting changes in strain to generate an electrical signal related to the clamping force. This process allows the clamping force to be measured directly and enables real-time monitoring and evaluation of the strain state of the tie rods.

**Figure 17:**
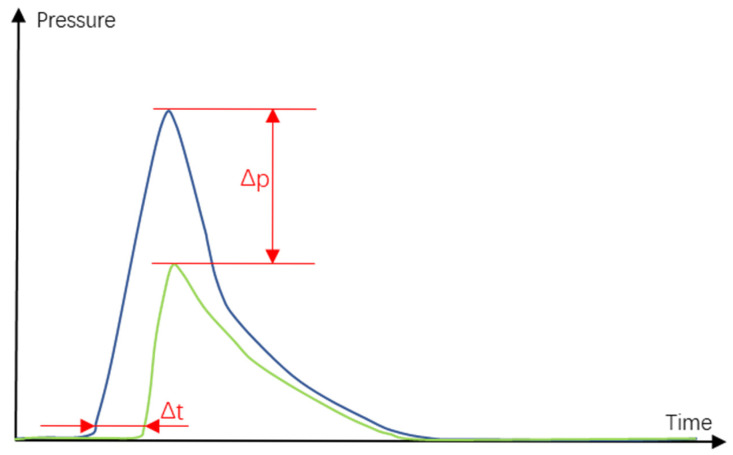
Cavity pressure curve during the injection molding cycle.

By monitoring the tie rod strain state in real time, Figure 17 is obtained, which allows effective control of the pressure in the mold to ensure the quality of the molded part during the injection molding process. Excessively high or low mold cavity pressure affects the quality of the final product, which may lead to dimensional deviations, surface defects, or degradation of the mechanical properties. With real-time feedback from strain sensors, operators can adjust the injection parameters in a timely manner to ensure that the pressure inside the mold remains within the optimal range, thus improving the overall quality and stability of the molded part. The following is the formula for the tie rod stress and clamping force:

$$\varepsilon_{i}=\frac{F_{i}}{EA}$$

$$F_{i}=\frac{EA\varepsilon_{i}\times 10^{9}}{9.81}$$

$$F=\sum_{i=1}^{n}F_{i}$$

where $\varepsilon_{i}$ represents the stress of the *i*th tie in Pa; *E* represents the Young’s modulus of the tie (=2.06 × 10^11^ Pa); *A* represents the cross-sectional area of a single tie in m^2^; *Fi* and *F* represent the *i*th tie and the total clamping force in tons, respectively; and *n* represents the number of ties.

In our analysis, as shown in Figure 17, for the curve obtained by the pressure sensor, The blue curve is high pressure and the green curve is low pressure, we also find that there is a close relationship between the node displacement, residual stress, and pressure in the mold cavity, which allows us to further explore the problem of large column elongation. The elongation of a large column is an important index in the injection molding process and affects the mechanical properties and stability of plastic parts. By optimizing the injection parameters, not only are the joint displacement and re-sidual stress increased but also the elongation of the large column is effectively controlled so that the plastic parts have better ductility and toughness under high-stress conditions.

## 6. Conclusions

Acoustic tweezers, sophisticated tools for non-contact micro-scale particle manipulation using sound waves, have extensive applications in biomedicine, like cell sorting and drug delivery; materials science, for nanomaterial assembly; and micro- and nanofabrication, in tasks such as micro-pattern transfer. However, their manufacturing process is intricate, with high-precision materials and process requirements that directly impact performance and stability. This research focuses on enhancing acoustic tweezer performance and reliability through injection molding process optimization.

We initiated this study by designing a 3D model of acoustic tweezers and comparing molds with 1, 2, and 4 cavities. The 4-cavity mold emerged as the best in terms of plastic flow. It is concluded that the design of four modes and four cavities performs best in terms of the plastic flow and filling effect. Subsequently, after evaluating various injection molding materials based on mechanical and thermal properties, PEEK was chosen as the base material.

In the optimization process, we apply the signal-to-noise ratio (S/N) evaluation method to convert the experimental results to evaluate the eigenvalues. By calculating the signal-to-noise ratio for 20 sets of data, we found that the optimized signal-to-noise ratio of nodal displacement was significantly improved. Ultimately, the optimal combination of injection parameters is determined by combining the simulation results and the recognizable performance evaluation, which significantly improves the acoustic tweezer performance.

1. The acoustic tweezer manufacturing process is further optimized by introducing IME technology. The application of IME technology not only improves the structural stability and functional reliability of the tweezers but also enhances the manufacturing efficiency and product consistency.

2. Three key injection molding parameters, holding pressure, holding time, and melting temperature, were optimized via the NSGA-II-MOPSO multi-objective hybrid optimization algorithm. The results show that the optimized injection molding process significantly reduces the nodal displacement, volumetric shrinkage, and residual stress, reflecting the effectiveness of the multi-objective optimization algorithm in tuning complex process parameters. The method provides a powerful solution for similar multiparameter and multi-objective optimization problems.

3. Using the signal-to-noise ratio evaluation method, we find that the signal-to-noise ratio of the optimized nodal displacement is significantly improved to 21.92 dB, which is significantly greater than that of 10.88 dB before optimization. This finding indicates that the performance and stability of the acoustic tweezers were significantly improved by optimizing the injection molding process parameters, which verifies the effectiveness and usefulness of the signal-to-noise ratio as an evaluation tool.

Through the systematic optimization and analysis in this study, we successfully enhanced the performance of acoustic tweezers and reduced the common defects in the production process, which verified the effectiveness of the NSGA-II-MOPSO and S/N ratio evaluation methods in practical applications. This study provides an important reference and methodological guidance for future injection molding process optimization and the development of high-performance acoustic devices.

## Figures and Tables

**Table 1:** Comparison of target values obtained by different cavities.

A Few Cavities in a Mold	1	2	4
Maximum warpage (mm)	0.502	0.514	0.491
Maximum volume shrinkage (%)	15.22	15.35	15.47

**Table 2:** The target values obtained by simulation.

Number	Holding Pressure (MPa)	Pressure Holding Time (s)	Melting Temperature (°C)	Node Displacement (mm)	Volume Shrinkage (%)	Residual Stress (MPa)
1	97.29	13.95	288.92	0.423	13.055	0.182
2	173.72	5.22	283.15	0.254	12.740	0.057
3	165.12	9.91	284.86	0.259	12.772	0.094
4	144.54	13.23	296.63	0.270	13.285	0.132
5	80.69	12.32	295.38	0.270	13.209	0.120
6	129.72	5.84	293.00	0.263	13.079	0.076
7	185.73	8.14	299.48	0.270	13.281	0.111
8	156.51	7.74	282.01	0.255	12.638	0.077
9	126.48	9.10	286.77	0.260	12.841	0.090
10	147.40	10.90	290.60	0.265	13.044	0.107
11	103.70	6.16	297.30	0.266	13.258	0.094
12	135.89	14.47	291.94	0.267	13.097	0.129
13	190.28	11.10	285.25	0.262	12.788	0.102
14	177.52	6.66	292.58	0.263	13.061	0.087
15	110.14	10.03	294.19	0.268	13.162	0.104
16	161.70	14.95	289.62	0.265	13.007	0.126
17	120.44	12.55	281.68	0.259	12.658	0.101
18	194.74	11.75	298.01	0.271	13.286	0.129
19	87.16	8.69	280.28	0.255	12.651	0.076
20	107.23	7.34	287.59	0.258	12.879	0.077
Avg	139.80	10.00	289.96	0.263	12.986	0.100

## Data Availability

The authors declare that the data supporting the results of this study are available in the paper. If any raw data files in other formats are required, they can be obtained from the corresponding author upon reasonable request.
